# Ligustrazine induces viability, suppresses apoptosis and autophagy of retinal ganglion cells with ischemia/reperfusion injury through the PI3K/Akt/mTOR signaling pathway

**DOI:** 10.1080/21655979.2021.1880060

**Published:** 2021-01-31

**Authors:** Hong-yan Du, Rong Wang, Jian-liang Li, Huang Luo, Xiao-yan Xie, Ran Yan, Yue-ling Jian, Jin-ying Cai

**Affiliations:** Department of Ophthalmology, The Affiliated Traditional Chinese Medicine Hospital of Guangzhou Medical University, Guangzhou, China

**Keywords:** Ligustrazine, ischemia/reperfusion injury, retinal ganglion cells, autophagy, PI3K/Akt/mTOR signaling pathway

## Abstract

Ligustrazine, an alkaloid monomer extracted from Chuanxiong Rhizoma, has the function of protecting nerve cells. However, the effect and mechanism of ligustrazine on retinal ischemia/reperfusion (I/R) injury still need to be clarified. In our study, retinal ganglion cells (RGC-5) were used to establish a retinal I/R injury model by anaerobic cultivation. Cell viability, autophagy, and apoptosis were evaluated by cell counting kit 8 assay, transmission electron microscopy, and TUNEL staining after treatment with ligustrazine, PI3K inhibitor Ly294002, and/or mTOR inhibitor rapamycin, respectively. Besides, the levels of PI3K/Akt/mTOR pathway and autophagy-related proteins were determined by western blot. Moreover, one-way ANOVA was adopted for inter-group comparisons of measurement data. Our results demonstrated that low-concentration ligustrazine significantly enhanced cell viability and suppressed cell autophagy and apoptosis of RGC-5 cells after I/R injury, suggesting the protective effect of low-concentration ligustrazine on retinal I/R injury. Moreover, the alleviating effect of ligustrazine on RGC-5 with retinal I/R injury was mechanistically associated with the activation of the PI3K/Akt/mTOR pathway. In conclusion, low-concentration ligustrazine has a significant protective effect on RGC-5 cells with retinal I/R injury by activating the PI3K/Akt/mTOR pathway.

## Graphical abstract

**Figure:**
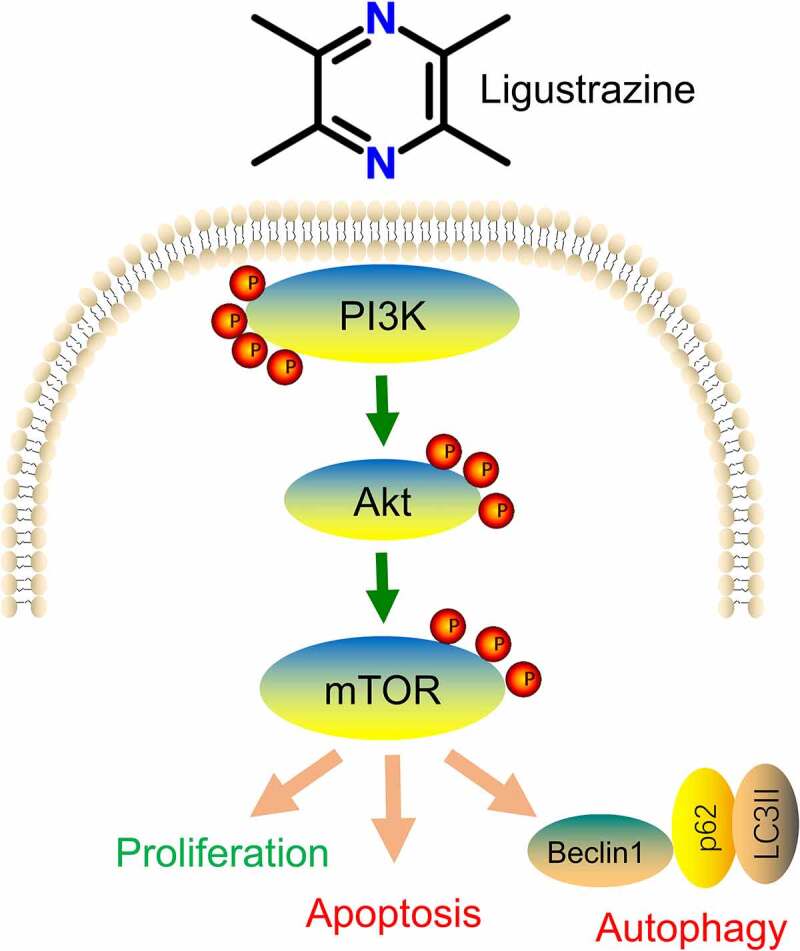

## 1. Introduction

Glaucoma is characterized by progressive neurodegeneration of optic nerve and loss of retinal ganglion cells (RGCs), which is the leading cause of irreversible blindness worldwide [1]. At present, glaucoma has severely affected the health of over 60 million people worldwide and has become the second leading cause of blindness [2]. Its pathogenesis is complicated but it is generally believed that the primary cause of glaucoma is the elevation of intraocular pressure (IOP) [3]. Therefore, lowering IOP has become the primary therapeutic method for treating glaucoma [4]. At present, various drugs and treatments have been applied to treat glaucoma by reducing IOP. However, they are not effective in preventing RGC death even when IOP is normalized [5,6]. Furthermore, the central retinal artery, as the terminal artery that supplies blood to the retina, is prone to ischemia and can cause severe retinal damage in a very short time period [7,8]. Reperfusion can aggravate reversible ischemic injury, resulting in different degrees of RGC death [9,10]. Currently, retinal I/R injury is a crucial pathogenesis of glaucoma [11,12]. Therefore, an in-depth exploration of the underlying mechanism of RGCs with retinal I/R injury is crucial for the diagnosis, treatment and prevention of glaucoma.

*Ligusticum chuanqiong* Hort, as a type of traditional Chinese medicine, has effects such as activating blood circulation, expelling congestion, and relieving pain [13,14]. Its primary active ingredient is ligustrazine (2,3,5,6-tetramethylpyrazine) [15]. Ligustrazine promotes blood circulation to remove blood stasis, halts platelet agglutination, expands arterioles, improves microcirculation, helps anti-oxidation, antagonizes calcium, and promotes anti-fibrosis [16,17]. In recent years, studies have reported that ligustrazine plays an important role in the craniocerebral vascular system, central nervous system, respiratory system, and digestive system; moreover, it has beneficial effects such as anti-cancer and anti-tissue fibrosis [18–20]. However, whether ligustrazine has a significant protective effect on retinal I/R injury remains unclear.

Autophagy is the process by which lysosomes degrade substances, and it has a remarkable effect on maintaining the stability of the intracellular environment by eliminating damaged cellular components [21]. Recent studies also have revealed that autophagy is associated with the pathophysiological process of glaucoma [22,23]. The phosphoinositide 3-kinase (PI3K)/Akt/mammalian target of rapamycin (mTOR) signaling pathway is one of the most crucial signal transduction pathways of intracellular autophagy, which inhibits cell apoptosis and promotes cell proliferation by affecting the activation state of various downstream effector molecules [24,25]. Therefore, we speculated for the first time that the PI3K/Akt/mTOR pathway may be the potential mechanism of ligustrazine affecting retinal I/R injury.

In our study, we established the RGC-associated retinal I/R injury model via hypoxia and reoxygenation cultivation. Then, we observed the entrainment of ligustrazine on the viability by cell counting kit 8 and the inhibition of ligustrazine on apoptosis and autophagy by TUNEL staining and transmission electron microscopy in RGCs with retinal I/R injury. Furthermore, we for the first time, we confirmed the activation of ligustrazine on the PI3K/Akt/mTOR pathway and the inhibition of ligustrazine on apoptosis-related proteins in RGCs with retinal I/R injury. Moreover, we confirmed the relationship between ligustrazine and PI3K/Akt/mTOR pathway via the rescued experiments in RGCs with retinal I/R injury. Therefore, our results proved that the PI3K/Akt/mTOR pathway is involved in the neuroprotective effect of ligustrazine on the I/R injury.

## 2. Materials and methods

### 2.1. Cell culture

RGCs (RGC-5, BNCC341515) were purchased from BeNa Culture Collection (Beijing, China) and grown in high-glucose DMEM (Gibco) containing 10% fetal bovine serum (FBS; Biochrom, S0615), 50 U/mL penicillin, and 50 g/mL streptomycin at 37.0°C in 95% air and 5% CO_2_.

### 2.2. Establishment of the I/R model

The RGC-5 cells were cultured in an anoxic incubator for 4 h to simulate an ischemic environment. Then, the cells were routinely cultured in an oxygen-saturated incubator with high-glucose DMEM containing 10% FBS for 24 h to construct an I/R model [26,27].

### 2.3. Cell treatment

RGC-5 cells in the logarithmic phase of growth were seeded in six-well plates and cultured in complete medium for 8 h until ~70% cell adhesion. I/R model cells were treated with 0, 5, 10, 20, 40, 80, 160, or 320 μg/mL ligustrazine (Baomanbio, D0188). Cultured I/R model cells were divided into I/R group [DMSO solution (Sigma, D2650)], ligustrazine group [10 μg/mL ligustrazine), ligustrazine+PI3K inhibitor group [10 μg/mL ligustrazine and 10 μM Ly294002 (TargetMol, T2008)], and ligustrazine+mTOR inhibitor group [10 μg/mL ligustrazine and 10 μM rapamycin (Solarbio, IR0010)]. In the normal (control) group, only an equivalent volume of DMSO was added to RGC-5 cells without hypoxia treatment. In addition to the RGC-5 cells in the normal group, the complete medium was replaced with low-glucose DMEM medium. RGC-5 cells were treated with ligustrazine and DMSO for 30 min, and the experiments were repeated five times.

### 2.4. Cell counting kit 8 (CCK-8) assay

RGC-5 cells in the logarithmic phase of growth (100 μL, 3000 cells/well) were seeded in a 96-well plate and incubated for 12 h at 37°C in a 5% CO_2_ humidified incubator. After these experimental treatments for 24 h, 10 μL of CCK-8 reagent (Abbkine, KTC011001) was added to each well, and the cells were cultured for 2 h in the incubator. Absorbance (OD_450_ value) was measured at 450 nm using a microplate reader (Infinite M200, Tecan, Austria), following which cell viability was calculated.

### 2.5. Transmission electron microscopy (TEM)

RGC-5 cells in each group were collected and fixed with 2.5% glutaraldehyde at 4°C for 2 h. After rinsing with phosphate-buffered saline (PBS) three times, the cells were fixed with 1% osmic acid at 4°C for 2 h. The cells were then dehydrated with a continuous gradient of ethanol and embedded in Embed-812 medium for 48 h. Ultrathin sections (60–80 nm) were prepared and stained with 2% uranium acetate and lead citrate for 15 min. Sections were then dried overnight at room temperature. Autophagosomes were also imaged using TEM.

### 2.6. TUNEL staining

Cells in each group were fixed with 3% paraformaldehyde (pH 7.4; 15710; Electron Microscopy Sciences, Hatfield, PA) at 4°C for 40 min according to the manufacturer’s instructions. After washing with PBS three times, 0.1% TritonX-100 and 0.1% sodium citrate solutions were added to penetrate at 4°C for 5 min. Cells were then washed again and treated with fluorescein-labeled nucleotide (dUTP) and TdT at 37°C for 60 min. Green fluorescent TUNEL-positive cells were analyzed using a fluorescence microscope.

### 2.7. Western blotting

After RGC-5 cells in each group were collected, they were rinsed with pre-cooled PBS three times and lysed with RIPA buffer (Sigma-Aldrich, R0278) containing a protease inhibitor on ice for 1 h. The supernatant was centrifuged at 12,000 × *g* for 15 min at 4°C. After the protein concentration was determined by the Pierce™ BCA Protein Assay Kit (23227; Thermo Scientific), total protein (30 μg) was separated by SDS-PAGE and transferred to a PVDF membrane (Millipore). The membranes were blocked with 5% skim milk powder at room temperature for 2 h and incubated with primary antibodies at 4°C overnight and secondary antibody (Abcam, ab199526) at room temperature for 2 h. Enhanced chemiluminescent reagent was used to develop images. The following primary antibodies were used: p-AKT (ab38449, Abcam), AKT (ab182729, Abcam), p-PI3K (ab127617, Abcam), PI3K (ab189403, Abcam), p-mTOR (ab131538, Abcam), mTOR (ab2732, Abcam), LC3 (ab48394, Abcam), Beclin-1 (ab207612, Abcam), SQSTM1/p62 (5114S, Abcam), and GAPDH (ab181603, Abcam).

### 2.8. Statistical analysis

Statistical analysis was performed using SPSS 18.0. All data were expressed as mean ± standard deviation. One-way ANOVA was performed for inter-group comparisons of measurement data. *P*-values of < 0.05 were considered to be statistically significant.

## 3. Results and discussion

### 3.1. Low-concentration ligustrazine protects RGCs after retinal I/R injury

**Figure 1.**
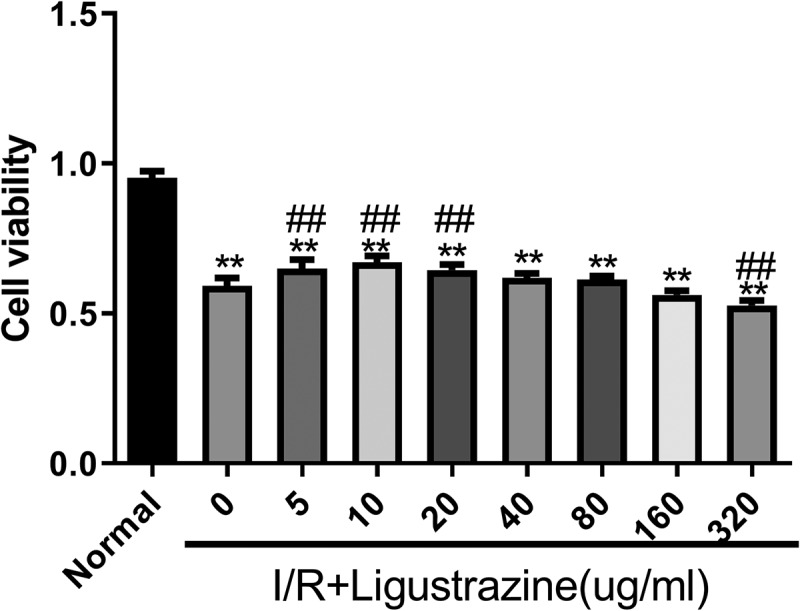
**Low-concentration ligustrazine had a protective effect on RGCs after retinal I/R injury**. After incubation with different concentrations of ligustrazine (0, 5, 10, 20, 40, 80, 160, or 320 μg/mL), CCK-8 assay was perfumed to verify the effect of ligustrazine on the cell viability of RGCs after retinal I/R injury. **p< 0.01 vs. normal group; ^##^*p*< 0.01 vs. model group

The retinal I/R injury is a frequent cause of blindness, and it mainly occurs in ischemic ophthalmopathy, particularly in glaucoma with intraocular hypertension [28]. Because of its high incidence, low cure rate, and poor treatment effect, retinal I/R injury has become a research hotspot worldwide. Therefore, it is of great importance to explore the mechanism underlying retinal I/R injury, and the injury model is the foundation of this research. In this study, we established an I/R injury model in RGCs. Moreover, we determined whether ligustrazine has protective effect on RGCs after retinal I/R injury. The results proved that cell viability was prominently reduced in the model group compared with that in the normal group, and I/R-induced reduction of RGC viability was dramatically reversed by low-concentration ligustrazine treatment, particularly by 10 μg/mL ligustrazine (*p*< 0.01, Figure 1). Thus, we suggested that I/R injury markedly inhibits the viability of RGCs, and this is reversed by low-concentration ligustrazine, indicating that low-concentration ligustrazine protects RGCs against I/R injury.

### 3.2. Inhibition of PI3K or mTOR reduced ligustrazine-mediated protection of RGCs after I/R injury

**Figure 2.**
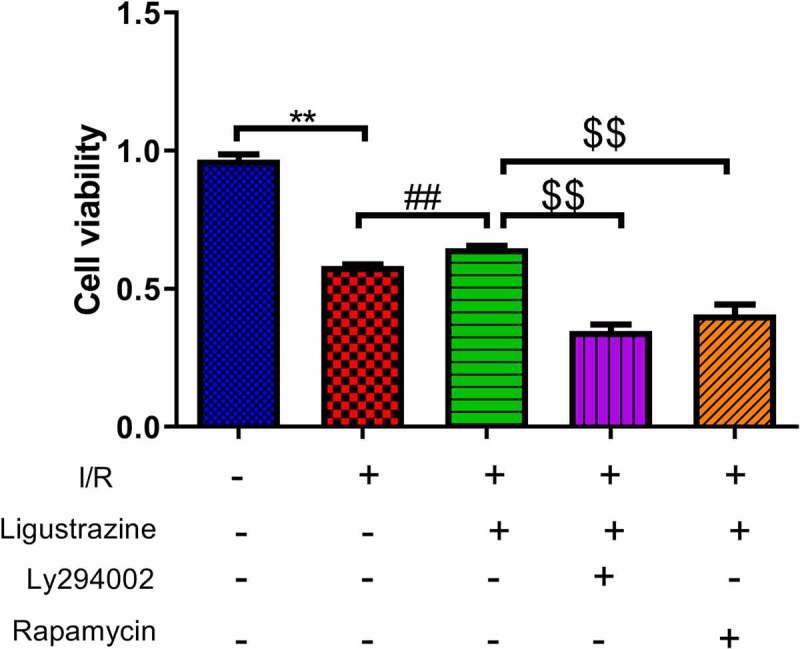
**Inhibition of PI3K or mTOR reduced ligustrazine-mediated protection of RGCs after I/R injury**. RGCs with retinal I/R injury were treated with 10 μg/mL ligustrazine alone or with 10 µM Ly294002 or 10 μM rapamycin. **p< 0.01 vs. normal group; ^##^*p*< 0.01 vs. model group. ^$$^*p*< 0.01 vs. ligustrazine group

The PI3K/Akt pathway is a signal transduction pathway involved in the regulation of cell growth, proliferation, and differentiation [25,29]. Akt is a serine/threonine protein kinase, and its activation can activate downstream factors such as the bcl-2 family to play an anti-apoptotic role [30]. Moreover, the activation of the PI3K/Akt pathway can phosphorylate Ser184 residues of Bax, thereby inhibiting apoptosis [31]. Previous studies have demonstrated that the PI3K/Akt signaling pathway is closely associated with RGC apoptosis induced by the retinal I/R injury [32,33]. mTOR is a relatively conserved serine/threonine protein kinase that participates in gene transcription and protein expression via the phosphorylation of its downstream target proteins, thus affecting biological activities such as autophagy and apoptosis [34,35]. Moreover, mTOR can depend on the PI3K/Akt pathway to participate in cellular biological activities [36,37]. Besides, researches testified that mTOR pathway is relevant to glaucoma [38,39]. However, it has not been clarified whether ligustrazine can play a protective role against retinal I/R injury by regulating the PI3K/Akt/mTOR pathway. To study this potential molecular mechanism of ligustrazine to protect RGCs against retinal I/R injury, PI3K inhibitor (Ly294002) or (mTOR inhibitor rapamycin) were applied in the cells. The results demonstrated that Ly294002 or rapamycin significantly reduced the viability of RGCs with I/R injury induced by ligustrazine (*p*< 0.01, Figure 2). Therefore, we concluded that inhibition of PI3K or mTOR reverses the promoting effect of ligustrazine on the cell viability of RGCs after I/R injury.

### 3.3. Inhibition of PI3K or mTOR reversed the decreased autophagy and apoptosis mediated by ligustrazine in RGCs induced by retinal I/R injury

**Figure 3.**
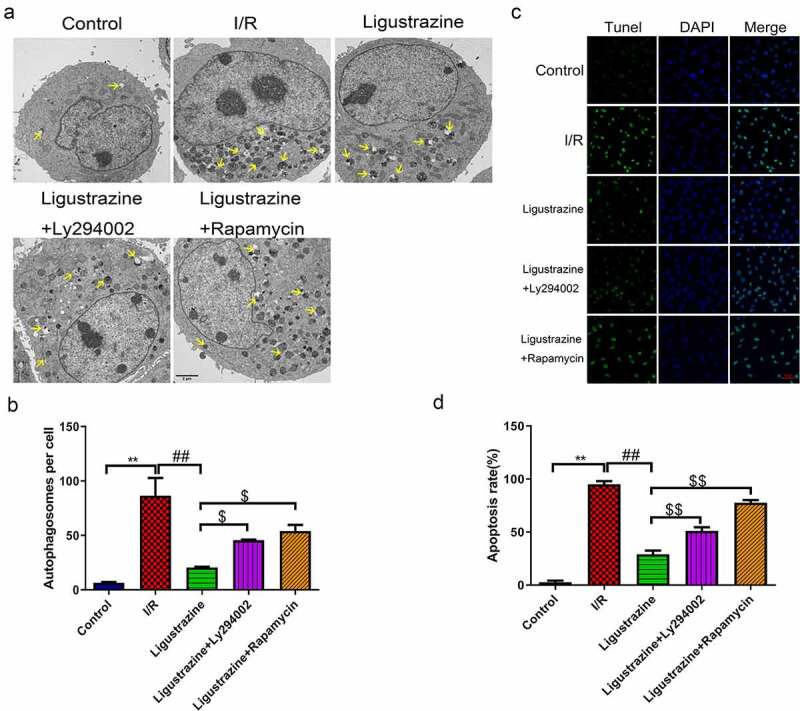
**Inhibition of PI3K or mTOR reversed the suppressed autophagy and apoptosis mediated by ligustrazine in RGCs induced by retinal I/R injury**. (a) After treatment with ligustrazine alone or ligustrazine with Ly294002 or rapamycin, the autophagy of RGCs after retinal I/R injury was identified by TEM. Yellow arrows indicate the number of autophagosomes. (b) The number of autophagosomes was counted, n = 3; **p< 0.01 vs. normal group; ^##^*p*< 0.01 vs. model group. $*p*< 0.05 vs. ligustrazine group. (c) Apoptosis was determined by TUNEL staining in the treated retinal I/R model. Magnification, 100 ×; Scale bar = 100 μm. (d) Apoptosis rate of the treated retinal I/R model RGCs was determined. **p< 0.01 vs. normal group; ^##^*p*< 0.01 vs. model group. ^$$^*p*< 0.01 vs. ligustrazine group

Previous studies have suggested that autophagy is closely related to the pathophysiology of glaucoma [22,23,40]. For example, miR-93-5p affects the NMDA-induced autophagy of RGCs through the AKT/mTOR pathway in glaucoma [39]. Furthermore, MEG3 induces the apoptosis of RGCs, which is related to the enhancement of autophagy [41]. Recent research has reported that the possible mechanisms of autophagy involved in the progression of glaucoma primarily include the removal of damaged mitochondria, the reduction of oxidative damage, inhibition of apoptosis, the inhibition of retinal microglia activation, and the reconstruction of damaged proteins and organelles [23]. In our study, we determined whether the inhibition of PI3K (Ly294002) or mTOR (rapamycin) affects the autophagy induced by retinal I/R injury of RGCs, which were mediated by low-concentration ligustrazine. As shown in Figure 3(a,b), the number of autophagosomes was prominently elevated in retinal I/R-injured RGCs, and this elevation was weakened by low-concentration ligustrazine treatment. Ligustrazine-mediated repression of autophagy of retinal I/R-injured RGCs was partially reversed by Ly294002 or rapamycin (*p*< 0.05). Therefore, we verified that the retinal I/R injury prominently accelerates RGC autophagy, which is reversed by the low concentrations of ligustrazine. Hence, the low concentration of ligustrazine protects RGCs from retinal I/R injury by blocking autophagy.

Apoptosis is one of the major mechanisms underlying the death of RGCs with retinal I/R injury and a common pathway of RGC degeneration [42]. Apoptosis is a type of programmed cell death cascade caused by a large number of death signal receptors under certain stimulus factors [43,44]. In our study, we observed that apoptotic cells in the model group were more numerous than those in the control group, and this apoptosis was observably attenuated by ligustrazine in RGCs after I/R injury. Ly294002 or rapamycin reversed the inhibitory effect of ligustrazine on the apoptosis of retinal I/R-injured RGCs (Figure 3(c,d)). This suggested that low-concentration ligustrazine notably weakens the elevated apoptosis that is induced in retinal I/R-injured RGCs.

In short, ligustrazine positively regulates the PI3K/Akt/mTOR pathway in RGCs with retinal I/R injury. Ligustrazine reduces autophagy and apoptosis in RGCs. Vice versa, the effect of ligustrazine on PI3K/Akt/mTOR is inhibited when RGCs are treated with Ly294002 and rapamycin.

### 3.4. PI3K/Akt/mTOR pathway participates in ligustrazine-mediated autophagy in RGCs induced by retinal I/R injury

**Figure 4.**
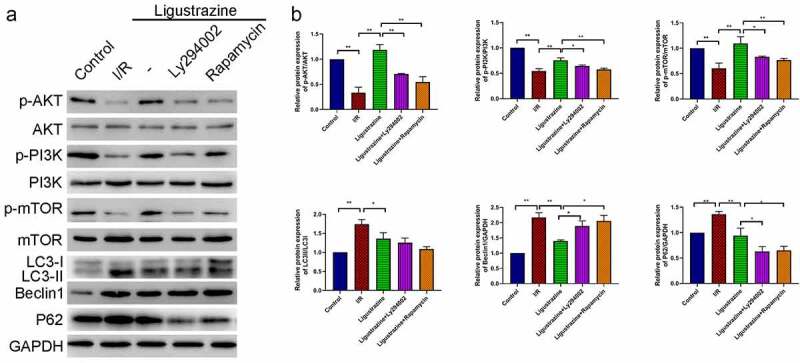
**PI3K/Akt/mTOR pathway participated in ligustrazine-mediated autophagy in RGCs induced by retinal I/R injury**. (a) After ligustrazine treatment, I/R-induced RGCs were treated with Ly294002 or rapamycin. Western blotting was performed to evaluate p-AKT, AKT, p-PI3K, PI3K, p-mTOR, mTOR, LC3, Beclin1 and P62 expression levels. (b) The relative expression levels of all proteins were calculated based on the gray values in each group. *p< 0.05, **p< 0.01; Gray values indicate relative expression levels of proteins

Next, we confirmed whether the PI3K/Akt/mTOR pathway plays a role in the protective effects of ligustrazine on I/R-injured RGCs. Our results certified that the expression levels of p-PI3K, p-Akt, and p-mTOR were dramatically reduced in RGCs after retinal I/R injury compared with that in normal RGCs, while the increase of p-PI3K, p-Akt, and p-mTOR expressions could be attenuated by low-concentration ligustrazine in RGCs after I/R injury; moreover, we uncovered that the upregulation of p-PI3K, p-Akt, and p-mTOR expressions that is mediated by ligustrazine could be prominently reversed by Ly294002 or rapamycin (*p*< 0.05, *p*< 0.01, Figure 4). These results suggested that Ly294002 or rapamycin could dramatically reverse the increase in p-PI3K, p-Akt, and p-mTOR expressions mediated by ligustrazine in RGCs after I/R injury. Therefore, the low concentration pf ligustrazine dramatically accelerates the phosphorylation of PI3K/Akt/mTOR pathway proteins in retinal I/R-injured RGCs. Subsequently, we discovered that LC3II/I, Beclin1 and P62 expressions were prominently increased in relative to that in normal RGCs, whereas low-concentration ligustrazine then significantly downregulated LC3II/I, Beclin1 and P62 in RGCs after retinal I/R injury; moreover, compared with the ligustrazine group, LC3II/LC3I, Beclin1, and P62 were significantly upregulated in the ligustrazine+PI3K inhibitor and PI3K inhibitor groups (*p*< 0.05, *p*< 0.01, Figure 4). Beclin1, LC3, and P62 are the three major proteins involved in autophagy and are the key indicators of the level of autophagy [45]. This suggests that inhibition of PI3K and mTOR reverses the protective effects of ligustrazine on autophagy in RGCs with retinal I/R injury.

In summary, we conclude that the low concentration of ligustrazine could induce the phosphorylation of PI3K, Akt, and mTOR to prevent autophagy of RGCs with retinal I/R injury.

## 4. Conclusions

Our results demonstrated that the introduction of PI3K inhibitor (Ly294002) or mTOR inhibitor (rapamycin) could reverse the enhancement of viability and suppression of autophagy and apoptosis of RGCs after retinal I/R injury, which were mediated by low-concentration ligustrazine, thereby uncovering a novel mechanism by which low-concentration ligustrazine protects the retina against I/R injury by activating the PI3K/Akt/mTOR pathway. Therefore, we suggested that appropriate ligustrazine might serve as a crucial protective agent for retinal I/R injury.

## Supplementary Material

Supplemental Material

## Highlights

- Ligustrazine induces the viability of RGCs after retinal I/R injury
- Inhibition of PI3K or mTOR attenuated ligustrazine-induced cell viability
- Ligustrazine inhibited the apoptosis and autophagy of I/R RGCs by PI3K or mTOR
- Ligustrazine activates PI3K/Akt/mTOR pathway
