# Supplementary figures and images for: Ligustrazine induces viability, suppresses apoptosis and autophagy of retinal ganglion cells with ischemia/reperfusion injury through the PI3K/Akt/mTOR signaling pathway
Source: Bioengineered. 2021 Jan 31;12(1):507–15. doi: 10.1080/21655979.2021.1880060 (PMC8806313; doi:10.1080/21655979.2021.1880060)

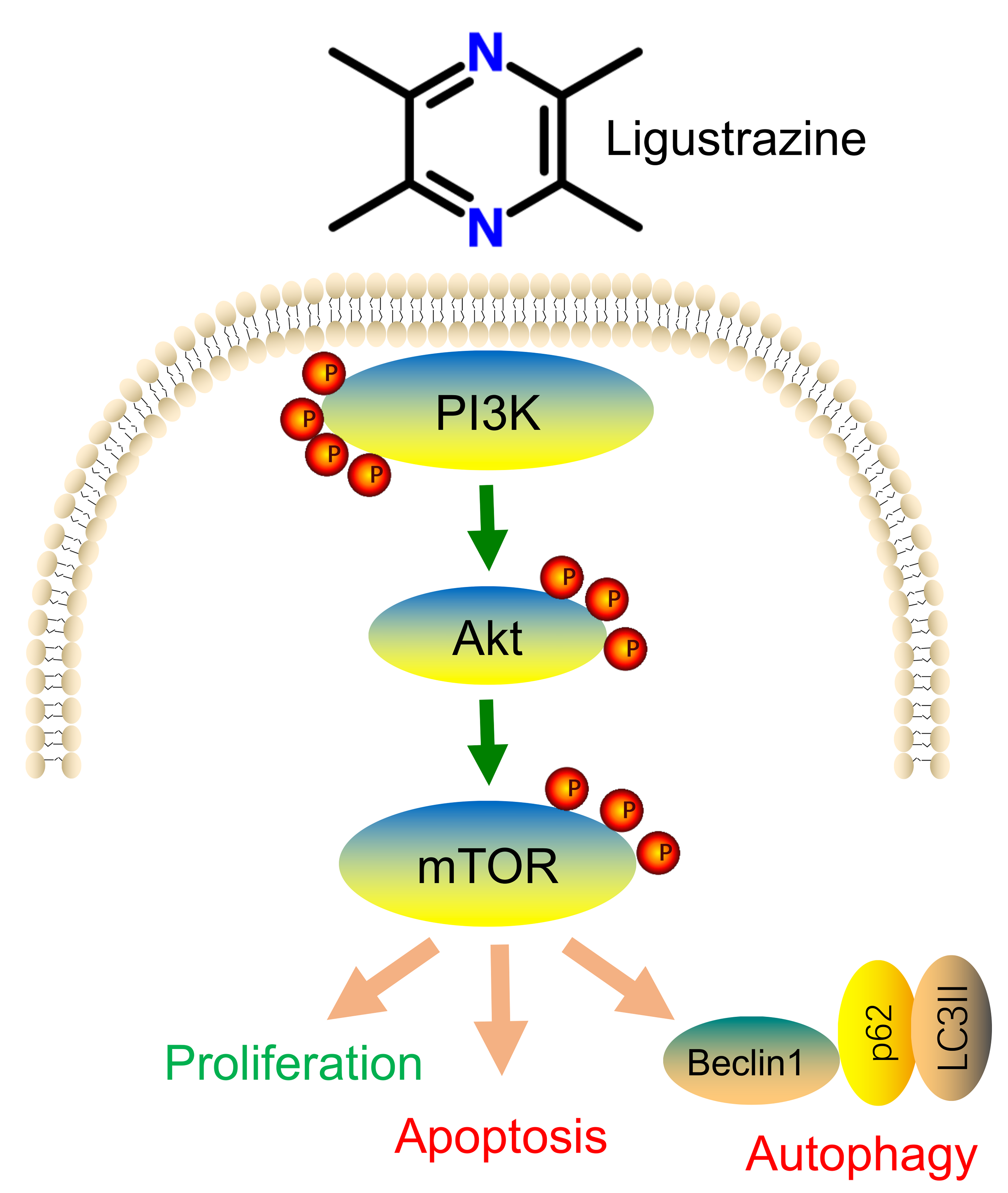

Supplement: Supplemental Material [file KBIE_A_1880060_SM2504.tif]
